# ﻿The Central American millipede family Rhachodesmidae in Costa Rica, with the description of a new species of *Aceratophallus* Carl, 1902 (Diplopoda, Polydesmida)

**DOI:** 10.3897/zookeys.1239.153272

**Published:** 2025-05-23

**Authors:** Sergei I. Golovatch

**Affiliations:** 1 Institute of Ecology and Evolution, Russian Academy of Sciences, Leninsky prospekt 33, 119071 Moscow, Russia Institute of Ecology and Evolution, Russian Academy of Sciences Moscow Russia

**Keywords:** Distribution, iconography, key, Neotropics, new record, taxonomy

## Abstract

The rather small millipede family Rhachodesmidae is endemic to Central America, and is represented in Costa Rica by only four species of the genus *Aceratophallus*, including *A.logunovi***sp. nov.** An illustrated key is given to all four species of Rhachodesmidae (= *Aceratophallus*) presently known to occur in Costa Rica. The first detailed iconography of a species of *Aceratophallus* is provided.

## ﻿Introduction

The millipede family Rhachodesmidae Carl, 1903 is endemic to Central America. Presently, it encompasses c. 63 species in 18 genera that range from Mexico in the north to Costa Rica in the south (Hoffman 1999; Hoffman et al. 2002; Enghoff et al. 2015). The genus *Aceratophallus* Carl, 1902 is among the largest in terms of species diversity, and its 12 currently accepted species range from Yucatán Peninsula in Mexico to Costa Rica (Hoffman 1999). According to Hoffman’s (1999) catalogue, which is still relevant and obviously requires no reiteration, only three species have so far been accepted as populating Costa Rica. The southernmost range periphery for both the family and the genus is as follows:

1. *Aceratophallusdux* Chamberlin, 1914, from Juan Viñas, Prov. Cartago, Costa Rica (Chamberlin 1914, 1922);
2. *Aceratophalluslamellifer* Brölemann, 1905, from San José (Brölemann 1905), recorded also from Parismina, Chitaria, and Tilarán, Costa Rica (Chamberlin 1922, 1933; Hoffman 1999);
3. *Aceratophallusunicolor* Carl, 1902, from San José, Costa Rica (Carl 1902), recorded also from El Gallito, Prov. Heredia or Alajuela, Costa Rica (Chamberlin 1922, 1933; Hoffman 1999).

The present note was prompted by the discovery of another new species of *Aceratophallus*, the first to be recorded in Costa Rica’s Guanacaste Province. An illustrated key is also provided to distinguish all four congeners that are known to occur in the country.

## ﻿Material and methods

The holotype of the new species treated below was collected in 2023 by Dmitry V. Logunov, Keeper of Arachnida and Myriapoda at the Zoological Institute of the Russian Academy of Sciences, St. Petersburg (ZISP), Russia. Colour photographs were taken at the Paleontological Institute, Russian Academy of Sciences (PIN), Moscow, using a Flexacam C1 camera mounted on a Leica M165С stereo microscope with built-in LasX software. Final image processing was performed with Adobe Photoshop CC. The distribution map was composed using QGIS ver. 3.32.1-Lima. Abbreviations used to denote particular structures of the gonopods are explained both in the text and figure captions.

## ﻿Taxonomy

### ﻿Class Diplopoda de Blainville in Gervais, 1844

**Order Polydesmida Latreille, 1802/03**

**Family Rhachodesmidae Carl, 1903**

#### ﻿Genus Aceratophallus

Taxon classificationAnimaliaPolydesmidaRhachodesmidae

Carl, 1902

3CC80904-C18A-5BD1-BC5B-41594E5FC2B2

##### Type species.

*A.unicolor* Carl, 1902, by original designation.

##### Diagnosis.

**Figures 1–11.**
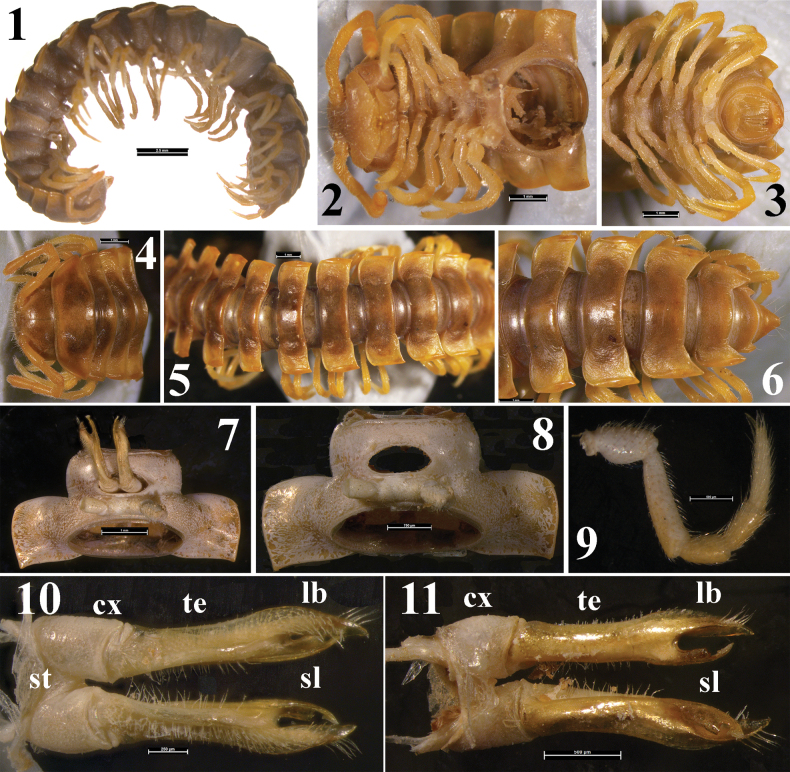
*Aceratophalluslogunovi* sp. nov., male holotype (ZISP MYR_DIP_0000189): **1** habitus, lateral view **2, 4** anterior part of body (head and rings 1–6), ventral and anterodorsal views, respectively **3, 6** posterior part of body, ventral and dorsal views, respectively **5** middle part of body, dorsal view **7, 8** body ring 7 with both gonopods in situ (7) and with both gonopods removed (8) **9** leg 9, lateral view **10, 11** both gonopods, ventral and dorsal views, respectively. Abbreviations: **cx** coxite. **lb** lateral branch, **sl** solenomere, **st** sternite, **te** telopodite. Photographs courtesy R.A. Rakitov.

Differs from other genera of the family primarily in the gonopodal telopodites (**te**) being densely setose all along their length, with its base being devoid of a clearly outlined spermal cavity/fossa (Fig. 14). With long and slender gonapophyses (**ga**) placed distomesally on male coxae 2 (Fig. 17). Slender, dorsoventrally flattened and only slightly diverging gonopods (Figs 1, 10, 11, 14–16, 20–22) directed cephalad and located inside a very simple, relatively small and transversely ovoid aperture (Figs 7, 8). Gonopods set centrally on a small triangular sternum (**st**) (Fig. 10). Each gonopod featuring a short cylindrical coxite (**cx**) (Figs 10, 11, 14, 18) and a longer, distally bipartite telopodite divided into a mesal solenomere (**sl**) and a lateral branch (**lb**) (Carl 1903). Like in all Rhachodesmidae, they are devoid of a cannula.

##### Composition.

Thirteen species, including the new one treated below.

Incorporation of the new congener described below into a key is straightforward, as all three *Aceratophallus* species from Costa Rica have long been keyed, albeit based solely on gonopodal characters (Attems 1940). The new key provided in the present contribution considers some somatic traits as well. Moreover, the distributions of all four Rhachodesmidae (= *Aceratophallus*) in Costa Rica have been mapped (Fig. 23).

#### ﻿ Aceratophallus logunovi sp. nov.

Taxon classificationAnimaliaPolydesmidaRhachodesmidae

D188472B-DD86-5D5F-9270-BA7FE3D1C53C

https://zoobank.org/4275A9F1-7667-4AC0-8702-FA354B98DDAB

Figs 1–11

##### Material examined.

***Holotype*** • male (in alcohol, ZISP MYR_DIP_0000189), Costa Rica, Guanacaste Province, entrance to Palo Verde National Park, tropical rainforest, 10°24'N, 85°19'W, 30.VI.2023, D. Logunov leg.

##### Name.

Honours Dmitry V. Logunov, the collector.

##### Diagnosis.

Differs from congeners primarily by the gonopodal telopodite being nearly straight, only slightly bent dorsad in basal 2/3 and erect in distal 1/3. With both solenomere (**sl**) and lateral (**lb**) branches pointed, held subparallel and closely attached to each other. The **sl** with a small lateral bulge clearly removed from the apex (see also key below).

##### Description.

Length 28 mm, width of midbody pro- and metazona 2.7 and 5.0 mm, respectively. General coloration in alcohol light brown with most of head, collum and following metaterga between paraterga faintly infuscate and brown; antennae, venter, legs, paraterga, central parts of collum and following metaterga and gonopods slightly lighter orange-yellow to yellow; legs and gonopods very faintly infuscate distad; calluses/peritremata on paraterga bright orange. Tegument generally smooth and shining; head and metazona, including sides, finely microgranulate; prozona shagreened and even more finely microgranulate. Sides of metazona below paraterga light greyish, a little more strongly granulate than prozona; strictures smooth; caudal halves of metaterga and, especially, bases of paraterga clearly and irregularly striolate (Figs 1–6).

Head very finely microgranulate all over; epicranial suture long, thin and fine, starting between antennae; clypeolabral region densely setose, vertex nearly bare; interantennal isthmus c. 1.5 times as wide as diameter of antennal socket (Figs 2, 4). Antennae only very slightly clavate, in situ extending past ring 4 dorsally (Figs 1, 4) (male). In length, antennomeres 2 = 6 > 3 > 4 = 5 > 1 > 7. Genae squarish.

In width, head < collum < ring 2 = 3 < 4 < 5–15, thereafter gradually tapering towards telson (Figs 4–6). Paraterga very well-developed (Figs 1–8) (male), always lying below level of a moderately convex dorsum, mostly set at about upper 1/4 midbody height, largely only poorly declined to subhorizontal. Collum sublanceolate, almost pointed at each end; both anterior and posterior margins straight medially; both anterolateral and lateral margins very broadly and regularly rounded and narrowly rimmed; paraterga poorly concave caudally (Fig. 4). Following paraterga with rimmed and faintly rounded to subrectangular anterior shoulders, each shoulder carrying a small denticle at anterolateral corner. Paratergal sides faintly rounded, fully taken up by lanceolate and narrow calluses/peritremata, these being always delimited by distinct sulci both dorsally and ventrally, clearly thicker and wider on poriferous rings than on poreless ones. Paratergal caudal corners first narrowly rounded and subrectangular, but starting with ring 3 increasingly acute, especially sharp and drawn past rear tergal margin on rings 13–18, but smaller, robust and sharp teeth on ring 19. Metatergal caudal margins narrowly rimmed, regularly and first faintly, but then increasingly concave (Figs 1, 4–6). Neither an axial line nor transverse metatergal sulci. Strictures between pro- and metazona faint and smooth. Limbus hyaline, thin, fine and microspiculate/microdenticulate. Pore formula normal (5, 7, 9, 10, 12, 13, 15–19). Ozopores dorsolateral, located near half to caudal 1/3 of poriferous paraterga inside oblong grooves. Pleurosternal carinae very small and rounded ridges visible only on rings 2–4. Spiracles inconspicuous. Epiproct as usual, rather long, finger-shaped, straight, subtruncate at tip, subapical setigerous knobs very faint (Figs 1, 3, 6). Hypoproct roundly subtriangular, with 1+1 indistinct rounded knobs removed from caudal margin (Fig. 3).

**Figures 12–22.**
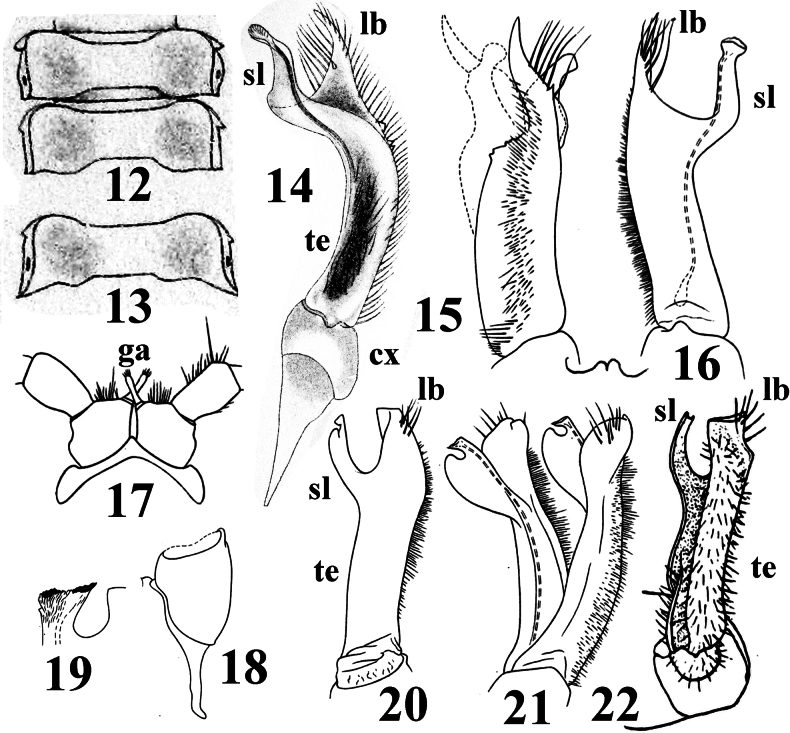
*Aceratophallusunicolor* Carl, 1902, ♂ syntype (12–16), *Aceratophalluslamellifer* Brölemann, 1905, ♂ syntype (17–21), and *Aceratophallusdux* Chamberlin, 1914, ♂ holotype (22): **12** two midbody rings, dorsal view **13** metatergum 17, dorsal view **14** left gonopod devoid of a cannula and with an elongated excavation instead of a basal fossa, mesal view **15, 16** right gonopod, ventral and lateral views, respectively **17** coxae and prefemora 2, caudal view **18** coxite of left gonopod, ventral view **19** tip of solenomere with a minute apical brush, mesal view **20** left gonopod, lateral view **21** both gonopods, dorsolateral view **22** left gonopod, ventral view. Abbreviations: **cx** coxite. **ga** gonapophyses, **lb** lateral branch, **sl** solenomere, **te** telopodite. **12–14**, after Carl (1902); **15–21**, after Brölemann (1905); and **22**, after Chamberlin (1914).

Legs long and slender, c. 1.7–1.8 times as long as midbody height (male) (Fig. 1), densely setose. In length, femur > tarsus > prefemur = postfemur = tibia > coxa. Tarsal claw small, simple and nearly straight (Figs 1–3, 9). Prefemora not bulged laterally (Fig. 9). Coxa 2 with a rather long, straight, coniform and apically fimbriate gonapophysis (male) (Fig. 2). Sterna densely setose, devoid of evident modifications. Tarsal brushes present only on a few anterior legs (Figs 2, 3).

Gonopodal aperture lanceolate, taking up entire central 1/3 prozonum 7, margins simple and not elevated (Figs 7, 8). Gonopods (Figs 7, 10, 11) in situ directed cephalad, typical of the genus, both only slightly diverging and located on a small triangular sternum (**st**). Coxites (**cx**) short, cylindrical and bare, c. 0.25 times as long as telopodites (**te**), each totally devoid of a cannula. Telopodites (**te**) slender, clearly flattened dorsoventrally, only slightly broadened distally, in situ basal 2/3 faintly and regularly curved dorsally, but distal 1/3 erect, only dorsal surfaces of **te** bare, all other sides densely setose throughout. Seminal groove starting inside a vague and oblong fossa near **te** base, but its course remaining clearly on mesal side. Distal third of each **te** branching into a somewhat shorter mesal solenomere (**sl**) and a longer lateral branch (**lb**). The **sl** carrying a minute apical brush and a small lateral bulge at its base, while **lb** an evident apical brush of longer setae before a sharp apex (Figs 10, 11). Both **sl** and **lb** pointed, held subparallel and closely attached to each other.

**Figure 23.**
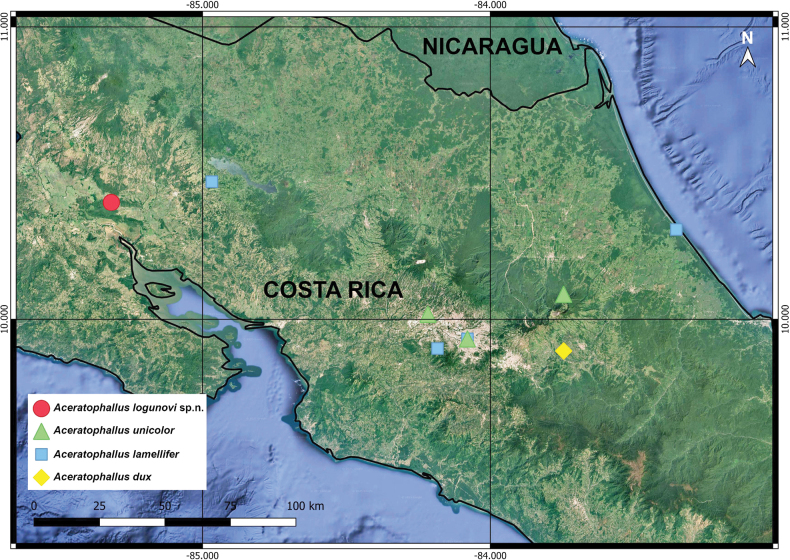
Distribution of Rhachodesmidae (= *Aceratophallus*) in Costa Rica.

### ﻿Key to *Aceratophallus* species in Costa Rica

**Table:** 

1(2)	Gonopodal telopodites (**te**) only slightly bent dorsad in basal 2/3, but erect in distal 1/3, both solenomere (**sl)** and lateral branch (**lb)** pointed, held subparallel and closely attached to each other, **sl** being much shorter than **lb** and supplied with a small lateral bulge clearly removed from apex (Figs 1, 10, 11)	***A.logunovi* sp. nov.**
2(1)	Gonopodal telopodites (**te**) regularly bent and broadened dorsad or entirely suberect, solenomere (**sl)** being clearly detached from lateral branch (**lb**)	**3**
3(4)	All paraterga below dorsum. Caudal corners/tips of paraterga slightly, but increasingly curved and directed laterad (Figs 12, 13). Gonopodal telopodites (**te**) with solenomere (**sl**) only a little longer than lateral branch (**lb**), **sl** being devoid of a small distal bulge, **lb** being acuminate (Figs 14–16)	***A.unicolor***
4(3)	Paraterga mostly upturned, with tips above dorsum. Caudal corners/tips of paraterga not curved laterad. Gonopodal telopodites (**te**) with solenomere (**sl**) about as long as lateral branch (**lb**), **lb** subtruncate to broadly rounded (Figs 20–22)	**5**
5(6)	A dark, interrupted, axial line on metaterga present. Hypoproct regularly rounded caudally. Solenomere (**sl**) particularly simple (Fig. 22)	***A.dux***
6(5)	A vague light axial stripe at most. Hypoproct subtriangular, narrowly rounded caudally. Solenomere (**sl**) with a small, subapical, mesal bulge (Figs 20, 21)	***A.lamellifer***

## Supplementary Material

XML Treatment for Aceratophallus

XML Treatment for Aceratophallus logunovi

## ﻿Additional information

### Data availability

All of the data that support the findings of this study are available in the main text.
